# AGE-RELATED CHANGES IN THE MUSCLE SECRETOME

**DOI:** 10.1093/geroni/igz038.378

**Published:** 2019-11-08

**Authors:** Ning Zhang, Susan Weintraub, Nicolas Musi

**Affiliations:** 1 Barshop Institute for Longevity and Aging Studies, University of Texas Health Science Center, San Antonio, Texas, United States; 2 Graduate School of Biomedical Sciences, University of Texas Health Science Center, San Antonio, Texas, United States

## Abstract

Skeletal muscle is one of the most abundant tissues in the body. In addition to its key roles in body support, movement and metabolic homeostasis, muscle also functions as an endocrine/secretory organ producing and releasing proteins into the circulation that modulate distant tissues (i.e. myokines). Considering that muscle mass and function changes with advancing age, here we tested the hypothesis that aging alters the muscle secretome profile. After euthanasia, soleus muscles from sedentary young and old mice were dissected, and incubated in oxygenated KRB buffer for 2 h. The buffer was subjected to in-gel trypsin-digestion and peptides analyzed by mass spectrometry. The concentration of 36 proteins were significantly (P<0.05) elevated in the young vs. the old group. In contrast, only 7 proteins were significantly elevated in the old group. Some notable differences include those in HSPA1B and HSPA5 that were detected only in the young group. HSPA8 also was significantly elevated by 1.8-fold (P<0.05) in the young versus the old group. Another prominent difference between groups involved translationally controlled tumor protein (TCTP), a critical regulator of apoptosis/carcinogenesis, that was elevated by 7-fold in the young vs. the old group (P<0.05). These results indicate that aging alters the muscle secretion profile. Identified differences in the muscle secretome could reflect intrinsic changes in muscle cells with age. Because these myokines are released into the circulation, it is also possible that myokine secretion is a regulated cellular process by which muscle communicates and modulates the aging process in distant tissues.

